# Epidemiology of Neuromyelitis Optica in the World: A Systematic Review and Meta-Analysis

**DOI:** 10.1155/2015/174720

**Published:** 2015-04-20

**Authors:** Masoud Etemadifar, Zahra Nasr, Behrang Khalili, Maryam Taherioun, Reza Vosoughi

**Affiliations:** ^1^Isfahan Research Committee of Multiple Sclerosis, Behesht Building Bozorgmehr Avenue, Isfahan 81588-44799, Iran; ^2^Medical Students' Research Center, Isfahan University of Medical Sciences, Isfahan, Iran; ^3^Health Sciences Centre, University of Manitoba, Winnipeg, MB, Canada R3A 1R9

## Abstract

*Background.* Neuromyelitis optica (Devic's disease) is a severe autoimmune inflammatory disorder of the central nervous system. Epidemiological aspects of NMO have not been systemically reviewed. In this study we systematically reviewed and assessed the quality of studies reporting the incidence and/or prevalence of NMO across the world. *Methods.* A comprehensive literature search using MEDLINE, EMBASE, and Web of Science for the terms “Neuromyelitis optica,” “devic disease,” “incidence,” “prevalence,” and “epidemiology” was conducted on January 31, 2015. Study quality was assessed using an assessment tool based on recognized guidelines and designed specifically for this study. *Results.* A total of 216 studies were initially identified, with only 9 meeting the inclusion criteria. High level of heterogeneity amongst studies precluded a firm conclusion. Incidence data were found in four studies and ranged from 0.053 per 100,000 per year in Cuba to 0.4 in Southern Denmark. Prevalence was reported in all studies and ranged from 0.51 per 100,000 in Cuba to 4.4 in Southern Denmark. *Conclusion.* This review reveals the gaps that still exist in the epidemiological knowledge of NMO in the world. Published studies have different qualities and methodology precluding a robust conclusion. Future researches focusing on epidemiological features of NMO in different nations and different ethnic groups are needed.

## 1. Introduction

Neuromyelitis optica (NMO), also known as Devic's disease, is a severe autoimmune inflammatory disorder of the central nervous system that can either present as a monophasic or relapsing disease that predominantly targets optic nerves and spinal cord [1]. Although NMO was described more than a century ago, there were few advances in understanding of the disease until discovery of NMO immunoglobulin G antibody (NMO-IgG) that led to better recognition of NMO patients with clinical signs and/or lesions in the CNS outside of the optic nerve and spinal cord [2, 3]. NMO has long been considered a subtype of Multiple Sclerosis (MS) due to the similarities between the clinical presentations of MS and NMO. It can be speculated that many NMO cases are never diagnosed and many others are misdiagnosed as MS. This might result in underestimation of prevalence and incidence of NMO [4]. Despite increasing literature about NMO epidemiology, prevalence and incidence rate in many countries have not yet been reported. Moreover, most of the available studies report regional rather than countrywide rates. In this study we aimed to systematically review the published epidemiological studies about prevalence and incidence of NMO in the world.

## 2. Materials and Methods

### 2.1. Selection of Studies

**Figure 1:**
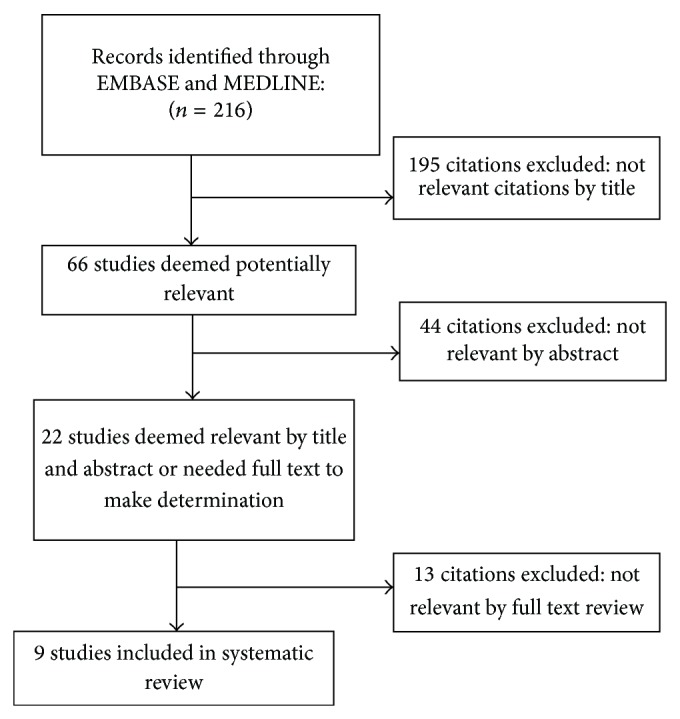
Flow diagram of selection of NMO incidence and prevalence studies within January 1, 1985–January 31, 2015.

A comprehensive literature search was performed using a search strategy developed by three authors with expertise in neurology, clinical epidemiology, and systematic review methodology (Masoud Etemadifar, Zahra Nasr, and Behrang Khalili). Both MEDLINE and EMBASE were searched for the terms “Neuromyelitis optica,” “devic disease,” “incidence,” “prevalence,” and “epidemiology” on January 31, 2015 (Figure 1). Review of Scopus and Google Scholar did not add any further results. Review articles and references in all papers were reviewed for potentially relevant studies.

### 2.2. Inclusion and Exclusion Criteria

The following criteria were used to select papers for inclusion in this systematic review:

1. NMO was defined according to accepted international diagnostic criteria (Wingerchuck criteria or Mayo Clinic criteria) [1, 5].
2. Prevalence of NMO was calculated.
3. Abstract of papers was published in English.

### 2.3. Review Methods

All duplicate records were removed and abstracts were screened by two reviewers (Masoud Etemadifar and Zahra Nasr) independently to assess their eligibility. Abstracts approved by at least one reviewer were deemed eligible for full text review. Complete copies of the potentially eligible studies were obtained and each study was reviewed independently by two trained reviewers (Reza Vosoughi and Zahra Nasr). Data were extracted by one reviewer using a standardized form comprising study location, dates of data collection, prevalence date or period, methods of case assessment and ascertainment, applied diagnostic criteria, and population study range. Crude and standardized (if available) prevalence and incidence rates were recorded for all reported regions, subgroups, and time periods. Extracted data were verified by a second reviewer.

### 2.4. Quality Assessment

Each of the two reviewers independently completed a quality review for each study to assess study eligibility for inclusion. Quality of studies was evaluated using an assessment tool designed specifically for this study based on a scoring system suggested by Boyle (Table 1) [6]. Quality of studies was scored out of 8 based on our scoring system composed of 8 questions. For studies based solely on registries, the reviewers were asked to mark “yes” for questions 3, 4, 5, and 6; and for studies using multiple sources of ascertainment, the reviewers were asked to mark “not applicable” for question 4, and quality was thus scored out of 7. A score of 8/8 or 7/7 was considered high quality while a score of 1/8 or 1/7 was considered low quality. A third reviewer was invited in case of lack of consensus between primary reviewers resulting in unresolved conflicts. All data abstraction and quality reviews were performed using the web-based DistillerSR program (Evidence Partners, Ottawa, ON, Canada). Meta-analysis was performed using Meta prop and Stata 11.2. Variance for each study was calculated using the binomial distribution formula. The presence of heterogeneity was determined by the chi-squared test with a significance level of <0.1 combined with an *I*_2_ statistic for estimates of inconsistency within the meta-analyses. The *I*_2_ statistic estimates the percent of observed between-study variability due to heterogeneity rather than to chance and ranges from 0 to 100 percent. (Values of 25%, 50%, and 75% were considered representing low, medium, and high heterogeneity, resp.). Each study prevalence estimate received a weight that was equal to the reciprocal of within-study variance (*v*_*i*_) summed with between-study variance (*τ*_2_).

## 3. Results

Nine articles reported the prevalence of NMO in different regions of the world (Table 3). The calculated tau-squared (*τ*_2_) or between-study variance for our analysis was 0.057. For this review we determined that *I*_2_ values above 75 percent were indicative of significant heterogeneity warranting analysis with a random effect model as opposed to the fixed effect model to adjust for the observed variability. Random effects models on the meta-analyses performed showed statistically significant heterogeneity [*I*_2_ = 97.1%, *p* < 0.001] (Table 2).

Incidence data were found in four studies and ranged from 0.053 per 100,000 per year in Cuba to 0.4 in Southern Denmark. Prevalence was reported in all studies and ranged from 0.51 per 100,000 in Cuba to 4.4 in Southern Denmark. Four of the studies presented the female/male ratio, all with a female preponderance varying from 2.27 : 1 in Isfahan, Iran, to 9.8 : 1 in French West Indies [7–11]. Six articles reported the mean age of onset [7–9, 11–13]. Isfahan, Iran, with the mean age of onset of 30 had the lowest and South East Wales with 39.5 had the highest mean age of onset [8, 9].

The oldest study published in 2008 from Mexico used Mayo Clinic criteria to identify 34 cases of NMO in Mexico City in the time period of 1993–2005 and reported a provisional prevalence rate of about 1/100,000 (the confidence interval has not been reported) [15]. There were two published studies in 2009 providing epidemiologic report about NMO [11, 12]. Cabrera-Gómez et al. applied Mayo Clinic criteria and collected data in 2003-2004 in Cuba mainland and reported prevalence rate of 0.52/100,000 (95% CI 0.39–0.67) and average annual incidence rate of 0.053/100,000 (95% CI 0.040–0.068). Results did not show a significant difference between three ethnic groups of the country: blacks, whites, and mulattoes (mixed). Cuba has the lowest rate of prevalence and incidence of NMO among these studies [12]. Another study in 2009 was published by Cabre et al. who reported a steady annual incidence of NMO of 0.2/100,000 (95% CI, 0.15–0.23) in the French West Indies for the period of 1992–2007 and a prevalence rate of 4.20/100,000 (95% CI, 3.7–5.7) in June of 2007 [11]. Next study is published from Denmark in 2011 and applied Wingerchuck criteria to assess the incidence and prevalence of NMO in the Region of Southern Denmark between 1998 and 2008. Forty-two NMO patients were identified. Prevalence rate was 4.4/100000 (95% CI 3.1–5.7) and incident rate was 0.4/100000 (95% CI 0.30–0.54). All patients were Caucasians except one. Southern Denmark represented the highest rate of prevalence and incidence in all nine studies [7]. In 2012 Cossburn et al. reported 14 Caucasian cases of NMO from South East Wales based on the review of registered neuroinflammation cases in University Hospital of Wales from 1985 to 2011 and calculated the prevalence rate of 2/100000 (95% CI: 1.22–2.97) in this region concluding that NMO in Northern European Caucasian population is as frequent as non-Caucasians [8]. The other study in 2012 was from Japan conducted in Tokachi province. With just three identified cases of NMO in 2011 fulfilling the 2006 Wingerchuk criteria, they calculated the prevalence rate of 0.9/100,000 (95% CI, 0.2–2.5) in Northern Japan; this region is considered a relatively low risk area for NMO [14]. In 2013 Jacob et al. published their epidemiologic data about NMO in adult population (age above 16) of Merseyside County of the United kingdom covering the time period of 2003–2010 [10]. The prevalence rate of 0.72/100000 (95% CI 0.31–1.42) and minimum combined average annual incidence rate of 0.08/100000 (95% CI 0.03–0.16) indicated that NMO is still an uncommon condition in UK adults. One year later in 2014, based on 2 reported cases of NMO and 9 reported cases of NMO Spectrum disorder, Pandit and Kundapur reported a prevalence rate of 2.6/100000 in Mangalore, south India, [13] for the study period from 2011 to 2013. In the latest published article in 2014, Etemadifar et al. identified 95 definite NMO cases in Isfahan, Iran, fulfilling Wingerchuk's criteria with overall crude prevalence rate of 1.9/100,000 (95% CI, 1.6–2.3) that is similar to other Caucasian populations [9].

## 4. Discussion

**Figure 2:**
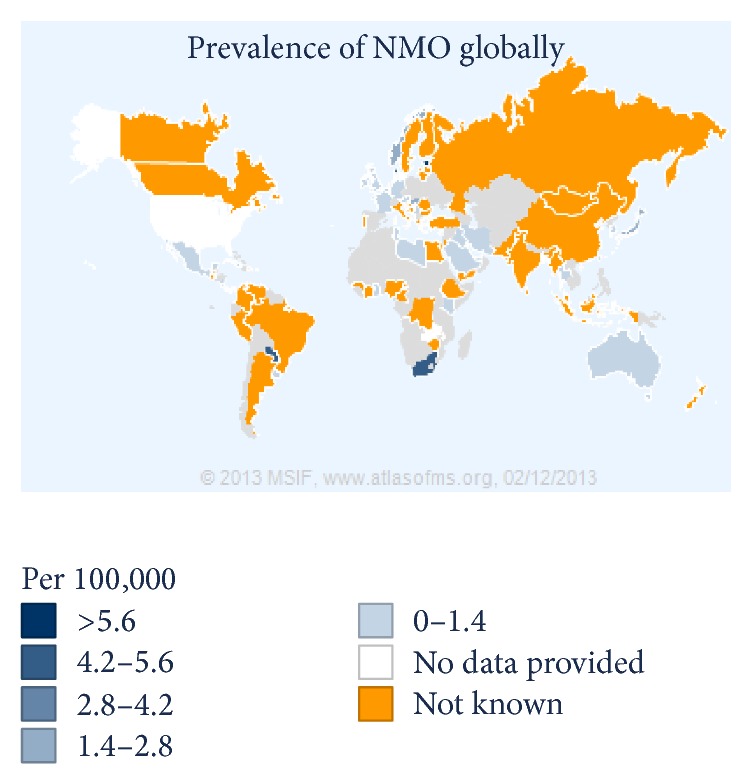
Prevalence of Neuromyelitis optica globally. (Data gathered from query data of Multiple Sclerosis International Federation (MSIF).)

Despite rapidly growing interests in NMO studies, the epidemiological studies about this disease are still sparse and our knowledge about the epidemiology of NMO in many parts of the world remains extremely limited.  Figure 2 and Table 4 are the estimation of NMO prevalence and incidence country by country gathered from query data of Multiple Sclerosis International Federation (MSIF) in 2013 [16]. In this review, we identified 9 articles worldwide reporting the prevalence of NMO [7–15], in which only one of them reported the country prevalence [12]. NMO prevalence in these studies varies geographically, from 0.51 per 100,000 in Cuba to 4.4 in Southern Denmark [7, 12]. Only four studies reported the incidence rate which was varying from 0.053 in Cuba to 0.4/100,000/year in Southern Denmark [7, 10–12]. Although the reason of these diversities is still unclear, different methodology, underestimation of the actual incidence, variable ethnicities of patients, and referral bias may be among the probable factors accounting for it. The prevalence of NMO might be more than what was reported as a number of cases were probably never diagnosed and some were misdiagnosed as MS.

Mayo Clinic criteria were applied in those studies which reported prevalence prior to 2006 [12, 15]. Seven of nine studies applied 2006 Wingerchuck diagnostic criteria and tested NMO-IgG [7–11, 13, 14]. Increasing recognition of NMO Spectrum Disorders and evolution of diagnostic criteria of the disease results in incidence reporting heterogeneity. Revised NMO diagnostic criteria proposed by Wingerchuk et al. in 2006 [17], which is the most recent diagnostic criteria of NMO and a modification of its predecessor [18], incorporated NMO-IgG positivity as one of the supporting pillars of diagnosis [1]. Four of nine articles in our review had reported NMO-IgG for their patients. Seropositivity for NMO-IgG was variable in these studies: 11/14 in South East Wales 11/14, 7/9 in the Merseyside county of United Kingdom, 3/3 in Northern Japan, and 63/95 in Isfahan, Iran [8–10, 14]. NMO-IgG is considered a highly specific test for NMO (85–100%) and lesser sensitivity (32–76%) [1, 7, 19]. Also different assay methods for NMO-IgG testing are different in terms of their sensitivity and specificity [20]. Amongst the reviewed articles only Asgari and Houzen reported their method NMO-IgG testing [7, 14]. The other articles had not described the assay method [8–10, 12, 13, 15].

One of the MS subtypes in Asian population especially in Japan is Opticospinal MS (OSMS) which differs from western type MS and is similar to NMO. Some of typical clinical and radiologic manifestations of NMO are common among Asian OSMS patients [21]. OSMS in Asian people demonstrate older age, female gender predominance, higher relapse rate, and more severe optic nerve and spinal cord involvement. NMO-IgG positivity in Asian OSMS is less frequent than in Western NMO [22]. A reevaluation of OSMS patients with application of 2006 Wingerchuk criteria and testing for NMO-IgG might help to clarify the identity of this ambiguous and probably heterogeneous group. Many patients with OSMS fulfill diagnostic criteria for NMO [1, 23, 24].

NMO cases have been reported from different regions of the world with various ethnicities. It had previously been suggested that NMO has ethnical predilection for nonwhites (10% of demyelinating disorders in Cuba versus 2% in countries with white population predominance) [12, 15, 22, 25–27]. About 15% to 57% of central demyelinating diseases in African–American, Japanese, and Indian populations were consistent with NMO while this disease comprised less than 2% of demyelinating diseases of the CNS in Caucasians [22, 25–27]. Interestingly, more recent studies suggest that prevalence of NMO in Caucasians is higher than what was previously believed [7, 8]. Cabrera-Gómez et al. reported prevalence rate of 0.426/100000 among whites and 0.691/100,000 in nonwhites in Cuba indicating lack of major difference in NMO prevalence in various ethnicities in Cuba [12]. However, further population-based studies encompassing larger populations are needed to evaluate the role of ethnicity in risk of developing NMO.

In conclusion, studies reported prevalence and incidence of NMO are mostly crude rates and these numbers are likely to rise due to increasing awareness of NMO and establishing diagnostic criteria to distinguish NMO from its mimickers. Retrospective nature of these studies might also contribute to biases in data collection. Future studies using single diagnostic criteria and longitudinal follow-up can help identifying temporal trends and geographic variations of the epidemiologic features of NMO in different regions of the world. Furthermore, variants of NMO spectrum disorders continue to be recognized. Better detection of NMO Spectrum Disorders in the future might change disease prevalence and incidence numbers. NMO registries following a consensus guideline about data collection and reporting and using single diagnostic criteria for NMO and NMO Spectrum Disorders might help to standardize epidemiologic reports about this uncommon condition. This will allow pooling all data and having a better understanding about global epidemiologic picture of NMO.

## Figures and Tables

**Table 1:** Quality assessment scores of multiple sclerosis incidence and prevalence studies.

Study (year)	Q1: target population described?	Q2: cases from entire population/probability sampling?	Q3: response rate >70%?	Q4: nonresponders clearly described?	Q5: sample representative of population?	Q6: data collection methods standardized?	Q7: validated criteria to assess disease?	Q8: were estimates given with confidence intervals?	Total score
Etemadifar et al. (2014) [9]	Yes	Yes	Yes	Yes	Yes	Yes	Yes	Yes	8/8
Pandit and Kundapur (2014) [13]	Yes	Yes	NR	Yes	Yes	No	Yes	No	5/8
Jacob et al. (2013) [10]	Yes	Yes	Yes	Yes	Yes	Yes	Yes	Yes	8/8
Houzen et al. (2012) [14]	Yes	Yes	Yes	Yes	Yes	Yes	Yes	Yes	8/8
Cossburn et al. (2012) [8]	Yes	Yes	NR	NA	Yes	Yes	Yes	Yes	6/7
Asgari et al. (2011) [7]	Yes	Yes	NR	NA	Yes	Yes	Yes	Yes	6/7
Cabrera-Gómez et al. (2009) [12]	Yes	Yes	NR	NA	Yes	Yes	Yes	Yes	6/7
Cabre et al. (2009) [11]	Yes	Yes	NR	NA	Yes	Yes	Yes	Yes	6/7
Rivera et al. (2008) [15]	Yes	NR	NR	NR	NR	No	Yes	No	2/8

NR: not reported; NA: not applicable; NC: not clear.

**Table 2:** Prevalence of NMO in studies with a random effect model.

Study	Prevalence	[95% Conf. interval]		% weight
		Lower	Upper	
Etemadifar et al. [9]	1.95	1.62	2.321	13.13
Pandit and Kundapur [13]	2.62	1.533	4.113	7.66
Jacob et al. [10]	1.14	0.675	1.846	12.01
Houzen et al. [14]	0.85	0.294	2.225	9.99
Cossburn et al. [8]	1.96	1.208	3.032	9.98
Asgari et al. [7]	4.41	3.456	5.413	9.53
Cabrera-Gómez et al. [12]	0.52	0.404	0.665	13.77
Cabre et al. [11]	4.2	3.336	5.116	10.09
Rivera et al. [15]	0.18	0.133	0.257	13.85
Pooled prevalence	1.82	1.265	2.365	100

Heterogeneity chi-squared = 277.51 (d.f. = 8);  *p* < 0.001.

*I*-squared (variation in ES attributable to heterogeneity) = 97.1%.

Estimate of between-study variance tau-squared = 0.057.

Test of ES = 0 : z = 6.46; p < 0.001.

**Table 3:** Prevalence and incidence studies of Neuromyelitis optica.

Study (year)	Region (subgroup)	Design	Case ascertainment	Prevalence day/period	Diagnostic criteria (established by)	Population denominated	Number of patients	Female/male ratio	Mean age		Prevalence per 100,000 (95% CI)	Incidence per 100,000 (95% CI)	Quality score
									Age of onset	Age of patients			

Etemadifar et al. (2014) [9]	Isfahan	Population-based	Administrative database	10/10/2013	Wingerchuck	4880430^∗^	95	2.27 : 1	30	36.6	1.9 (1.6–2.3)		8/8

Pandit and Kundapur (2014) [13]	Mangalore		3 medical social workers	1-2011 to 6-2013	Wingerchuk	419,306	11			40 ± 18	2.6		5/8

Jacob et al. (2013) [10]	Merseyside		Administrative database	30/12/2010	Wingerchuk	1,145,322	13	3.5 : 1			0.72 (0.31–1.42)	0.08 (0.03–0.16)	8/8

Houzen et al. (2012) [14]	Tokachi		10 MS-related institutions	31/3/2011	Wingerchuk	352,353	3				0.9 (0.2–2.5)		8/8

Cossburn et al. (2012) [8]	South East Wales		Regional neurologist and hospitals & administrative database	01/05/2010	Wingerchuck	712,572	14	4.5 : 1	39.5	49	2 (1.22–2.97)		6/7

Asgari et al. (2011) [7]	Southern Denmark	Population-based	4 neurology and 3 ophthalmology departments	1/1/1998–31/12/2008	Wingerchuck	952,000	42	2.8	35.6		4.4 (3.1–5.7)	0.4 (0.3–0.54)	6/7

Cabrera-Gómez et al. (2009) [12]	Cuba		Neurologists, hospitals, administrative database	30/11/2004	Mayo Clinic	11,177,743	58		31.8	41.7	0.519 (0.394–0.671)	0.053 (0.040–0.068)	6/7

Cabre et al. (2009) [11]	French West Indies	Population-based	Neurologists, hospitals, administrative database	June 2007	Wingerchuck	1142857^∗^	48	9.8	30.9	39.8	4.2 (3.7–5.7)	0.20 (0.05–0.35)	6/7

Rivera et al. (2008) [15]	Mexico City		One tertiary care referral center	1993 to 2005	Mayo Clinic	18,400,000	34				1		2/8

^∗^Calculated from available data in the report.

**Table 4:** Prevalence of Neuromyelitis optica globally (data gathered from query data of Multiple Sclerosis International Federation (http://www.msif.org/)).

Number	Country	Number of people with NMO	Prevalence of NMO (per 100,000)	Incidence of NMO (per 100,000)	Mean age of NMO onset
1	Albania	25	1.3		
2	Australia	18	0.08		33
3	Austria	71	1		45.7
4	Bahrain	10	2	2	25
5	Belgium	25	0.23		
6	Costa Rica	18	0.4		
7	Cuba	58	0.52	0.05	
8	Cyprus	10	1.1	0.2	33.1
9	Denmark	200	4.4	0.4	35.6
10	Estonia	105	7		
11	France	325	0.5	0.2	31
12	Germany	1050	1.3		39
13	Ghana	30	0.12		30
14	Hungary	140	1.41		
15	Islamic Republic of Iran	1000	1.29		32
16	Iraq	16	0.05	0.0015	32.5
17	Ireland	50	1		35
18	Japan	3500	2.75		37
19	Kenya	20	0.05		
20	Kuwait	15	0.45		29
21	Libya	5	0.08		
22	Malta	1	0.23		60
23	Mexico	1000	1	0.12	30
24	Netherlands	500	5		35
25	Nicaragua	10	0.2	0.04	
26	Norway	80	2	0.2	35
27	Paraguay	122	5	0.8	30
28	Republic of Korea	420	0.85		34
29	Saudi Arabia	20	0.07		30
30	Serbia	45	0.47		41.3
31	Singapore	144	2.66		44
32	South Africa	400	5	1	35
33	Taiwan	400	1.72		
34	Thailand	286	0.43		37
35	Tunisia	10	0.09		
36	United Arab Emirates	15	0.16		35
37	United Kingdom	400	0.7	0.2	40.6
38	Uruguay	20	1		30
